# Applying principles to practice: cleaning and disinfection of extended reality equipment used in healthcare settings

**DOI:** 10.1017/ice.2025.10249

**Published:** 2025-08

**Authors:** Thomas S. Murray, Erica S. Shenoy, Scott C. Roberts, Richard Martinello, Asher Marks, Kimberly Hieftje, Chloe V. Green, Joseph T. Rothschild, Morgan Shradar, Tess McKinney, Lindsey Jo Hand, Shannon Novosad, Janet Glowicz

**Affiliations:** 1 Department of Pediatrics, Yale School of Medicine, New Haven, CT, USA; 2 Department of Laboratory Medicine, Yale School of Medicine, New Haven, CT, USA; 3 Massachusetts General Hospital and Mass General Brigham, Boston, MA, USA; 4 Harvard Medical School, Boston, MA, USA; 5 Department of Internal Medicine, Yale School of Medicine, New Haven, CT, USA; 6 Division of Laboratory Systems, Training and Workforce Development Branch, United States Centers for Disease Control and Prevention, Atlanta, GA, USA; 7 Nebraska Medicine, Omaha, NE, USA; 8 Department of Pathology, University of Nebraska Medical Center, Omaha, NE, USA; 9 Division of Healthcare Quality Promotion, Health Systems Strengthening Resilience Training Branch, United States Centers for Disease Control and Prevention, Atlanta, GA, USA

## Abstract

The use of extended reality (XR) for education of healthcare personnel (HCP) is increasing. XR equipment is reusable and often shared between HCP in clinical areas; however, it may not include manufacturer’s instructions for use (MIFU) in healthcare settings. Considerations for the selection of equipment and development of cleaning and disinfection protocols are described.

## Highlights

- The use of extended reality in healthcare is increasing.
- MIFU for cleaning and disinfection in healthcare settings may be absent.
- Consider consulting local subject matter experts for standardized cleaning and disinfection processes.

## Introduction

Extended reality (XR) encompassing augmented reality, virtual reality (VR), mixed reality, and other immersive computer-generated realties has become increasingly used in training and education within healthcare.^1^ XR technologies create engaging learning experiences that enable healthcare personnel (HCP) to demonstrate understanding of concepts through gamification and immersive, realistic implementation of skills.^2^ Given the versatility, scalability, potential to enhance learning outcomes, and possible cost-effectiveness of XR equipment and simulation programs, healthcare education programs will likely continue to expand the use of these technologies. Beyond their use by HCP, there has been increasing interest and application of use of this technology, including use of XR training, by patients and their family members.^3^

XR programs are delivered on a wide variety of devices, including wearable head-mounted devices accompanied by hand controllers, touch screens, and computer keyboards to perform simulations.^1^ When the device is used in healthcare settings solely for training purposes and not for diagnosis or delivery of treatment, components of XR hardware are not considered medical devices.^4^ When used in clinical care areas, like inpatient units, to reduce the risk of direct or indirect contact with contaminated XR equipment, appropriate cleaning and disinfection between uses is necessary. This article provides considerations for selecting and maintaining XR equipment used in healthcare settings aligned with existing guidelines from the Centers for Disease Control and Prevention (CDC) for cleaning and disinfection.^5^

## Potential transmission risks associated with the use of XR equipment

XR equipment is reusable and can be shared among users in patient or resident care spaces. Equipment used for XR training may include complex surfaces like hand controllers with switches and joysticks. XR training developed in partnership with CDC’s Project Firstline have been designed for trainings conducted in clinical areas of inpatient units and by family members of high-risk critical care patients. When not appropriately cleaned and disinfected, XR equipment may facilitate transmission of pathogens between HCP and potentially to patients through direct and indirect contact. Despite earlier social media reports, no healthcare-associated transmissions have been published specifically related to XR equipment.^6^ However portable electronic devices can become highly contaminated with bacterial and viral pathogens in patient care environments.^7^ Previous assessments of the cleaning and disinfection of XR equipment have reported that local infection control (IC) experts were not involved in performing risk assessments and that standard operating procedures for the cleaning and disinfection of XR equipment were absent.^8^

### Principles

When selecting equipment and developing cleaning and disinfection protocols for XR equipment, consideration should be given to the manner in which existing IC principles, including Standard Precautions, such as hand hygiene and cleaning and disinfection of equipment between uses will be implemented.^9^ Whether training takes place within or outside of clinical areas, HCP with specific illnesses, such as conjunctivitis, should be excluded from using XR equipment just as they would be restricted from patient care.^10^ These same exclusions should apply to patients and family members with suspected illnesses. Additionally, XR equipment used for training can be segregated from that used for medical care or treatment.

### Involvement of local infection control experts

We recommend involving local IC subject matter experts in selecting and introducing the equipment into healthcare settings and ensuring cleaning and disinfection processes that HCP can carry out effectively.

### Selection of equipment

Equipment should be evaluated for the presence of porous surfaces and compatibility of nonporous surfaces with disinfectants.^8^ Equipment with nonporous surfaces is preferred. If equipment with porous surfaces cannot be avoided, then dedicating equipment to a single user or providing barrier coverings (eg, disposable face pads) should be considered.^8^

### Selection of products for cleaning and disinfection

Though intended for medical devices, the Spaulding Classification places XR equipment in the category of noncritical devices that should be cleaned and disinfected with low to intermediate-level United States Environmental Protection Agency (EPA) registered disinfectants.^5,11^ Criteria for selection of cleaning and disinfection products for use with XR equipment should include effectiveness against pathogens of interest, manufacturer’s instructions for use (MIFUs), compatibility with the equipment, feasibility of adherence to all disinfectant product label instructions, and HCP safety. Effective disinfection of nonporous surfaces of VR equipment has been demonstrated using either 70% isopropyl alcohol or alcohol-free quaternary ammonium wipes; however, any EPA-registered disinfectant with kill claims as permitted by the facility can be considered.^8,11^ Cleaning must be done prior to disinfection; therefore, if the product does not specify cleaning and disinfection in one step, two products (ie, a cleaning agent and a disinfecting agent) are needed. If EPA-registered disinfecting agents effective against organisms of interest (eg, EPA List B, H, P)^11^ are incompatible with MIFU of the XR equipment or if there is contact with mucous membranes, defer use of the equipment and consult the manufacturer and IC for assistance to determine the appropriate disinfection and cleaning methods. It is also important to document how the methods were chosen.

### Consideration of no-touch disinfecting methods

At this time, neither the EPA nor the CDC makes recommendations about no-touch devices, like Ultraviolet (UV-C)-technology, for the disinfection of XR equipment. If the manufacturer provides or recommends no-touch disinfection, determine whether this should be considered an adjunct to manual processes; use of such technologies does not obviate the need for manual cleaning to remove any bioburden prior to disinfection. The UV-C bulbs should be compatible with the surfaces of XR equipment used in healthcare settings, and methods of validating no-touch disinfection. As with all disinfectants, to ensure safe operation, HCP must follow the MIFUs of no-touch technology.

### Cleaning and disinfection of XR equipment

It is recommended that cleaning and disinfection of equipment used in healthcare settings be performed by trained HCP, including when used by patients or family members.^5^ In addition to supplies for cleaning and disinfection, personnel should have ready access to supplies for hand hygiene, and personal protective equipment (PPE), such as gloves if indicated, to prevent contact with chemical disinfectants. Following each use of the equipment, hands should be cleaned, and surfaces of the devices, including strap, casing, inner and outer facepieces, controllers, and all other surfaces, should be cleaned and disinfected. If disinfecting wipes are used, ensure adequate coverage of surfaces, so that they remain wet for the disinfectant contact times as indicated on the product label. The number of devices that can be cleaned with each wipe may vary according to the product label, complexity, and soiling of the equipment. Personnel performing cleaning and disinfection should be aware of indications for the use of new wipes.

### Storage of equipment after cleaning and disinfection

Once cleaned and disinfected, XR equipment would best be securely stored in a clean and dry location separate from items that have not been cleaned and disinfected.^5^ Disinfectants may degrade equipment in an accelerated manner. Prior to each use, it is recommended to assess XR equipment for damage (eg, cracks) that would hinder effective cleaning and disinfection. If damage is noted, the equipment should be considered for removal from use.

## Conclusion

XR simulation programs for training HCPs, patients and family members are increasingly prevalent.^1^ Because equipment used in XR education and training are not considered medical devices, standardized processes for cleaning and disinfection specific to healthcare settings are needed. Local IC experts can advise on equipment selection, and cleaning and disinfection protocols.
